# Relations between sweetened beverage consumption and individual, interpersonal, and environmental factors: a 6-year longitudinal study in German children and adolescents

**DOI:** 10.1007/s00038-020-01397-0

**Published:** 2020-06-20

**Authors:** Sven Schneider, Jutta Mata, Philipp Kadel

**Affiliations:** 1 Division Head Child and Health, Medical Faculty Mannheim, Mannheim Institute for Public Health, Social and Preventive Medicine, Heidelberg University, Ludolf-Krehl-Straße 7-11, 68167 Mannheim, Germany; 2 Health Psychology, University of Mannheim, 68161 Mannheim, Germany

**Keywords:** Soft drinks, Soda, Obesity, Individual factors, Interpersonal factors, Environmental factors

## Abstract

**Objectives:**

This study aims to characterize the consumption of sweetened beverages (SB) among young people in Germany in terms of individual and interpersonal-environmental correlates.

**Methods:**

A representative sample of children and adolescents from Germany was assessed twice, 6 years apart (total longitudinal sample *n* = 11,691 children and adolescents aged between 0 and 17 years old; weighted). The relations between individual and interpersonal-environmental factors at baseline with SB intake 6 years later were analysed using bivariate and multivariate methods.

**Results:**

The majority of children and adolescents in Germany consume sweetened beverages weekly, 23% daily. SB consumption is particularly high in boys and often accompanied by other unhealthy lifestyle behaviours including a high level of tobacco and media consumption with a concurrent deficiency in fruit and vegetable consumption. Interpersonal factors associated with higher sweetened beverage consumption include low socio-economic status, tobacco consumption of parents, and older maternal age.

**Conclusions:**

Research on factors that correlate with sweetened beverage consumption is crucial to design effective interventions. Our findings underline the importance of complex, multi-level interventions to target sweetened beverage intake and obesity.

**Electronic supplementary material:**

The online version of this article (10.1007/s00038-020-01397-0) contains supplementary material, which is available to authorized users.

## Introduction

Effective promotion of healthy eating is central to fighting the growing global obesity epidemic. Although reverse causality always needs to be considered, the majority of primary studies, reviews, and meta-analyses conclude that sugar-sweetened beverages (SSBs) intake causes excess weight gain. They suggest that limiting SSB consumption will significantly reduce the prevalence of obesity, particularly among children and adolescents (Keller and Bucher Della Torre 2015). Longitudinal data show that even one additional serving of SSBs per day is associated with greater risk of increased BMI among adolescents (Bogart et al. 2017). This might be because drinking SSBs does not lead to feelings of satiety, but rather increases hunger levels and prompts food consumption (Bogart et al. 2017; Hennessy et al. 2015). In addition to its role in obesity development, excessive consumption of SSBs has consistently been associated with the development of metabolic syndrome, type 2 diabetes, poor oral health (e.g. dental caries), and the displacement of milk and calcium in the diet (Garnett et al. 2013; Mazarello Paes et al. 2015).

The World Health Organization has therefore emphasized the “need to evaluate different behavioural-change approaches to promote the reduction of free sugars intake; in particular the intake of sugar-sweetened beverages (…) especially among children” (WHO 2015). Because many unhealthy dietary habits, such as SSB consumption, are formed during childhood, research on factors that influence these behaviours in children and adolescents is of utmost importance (Mazarello Paes et al. 2015). Understanding the relative importance of factors that correlate with SSB consumption is an essential prerequisite to identify and prioritize those with the greatest potential to reduce SSB consumption (Pettigrew et al. 2015) and to inform effective intervention development. This is the starting point for our study. We consider both that nutrition only constitutes one part of the health-relevant aspects of an individual’s lifestyle and that this lifestyle is the result of social contexts and environments. In this study, we therefore follow Mazarello Paes et al.’s (Mazarello Paes 2015) suggestion to split the potential correlates of sweetened beverages consumption into individual and interpersonal-environmental variables.

This study aims to characterize the consumption of sweetened beverages among young people in Germany in terms of individual and interpersonal-environmental correlates. The majority of studies conducted on this topic tend to be small, non-representative cohorts with a cross-sectional design (Mazarello Paes et al. 2015). In contrast, the analysis presented here is based on a large-scale nationwide representative dataset featuring a longitudinal design which allowed consideration of correlates at baseline and of the main outcome 6 years later.

## Methods

### Data

The following analyses are based on the “German Health Interview and Examination Survey for Children and Adolescents” (“KiGGS”). This longitudinal study is the first official database to generate comprehensive health data for cohorts between the ages of 0 and 17 in Germany. It was commissioned as the largest long-term study on children and adolescents in Germany by the Federal Ministry of Health and is managed by the Robert Koch Institute, Berlin (Hölling et al. 2012). To guarantee the study’s representativeness, a stratified multi-stage probability sample was selected from the official registers at local residents’ registration offices. The fieldwork for the baseline survey was carried out in 167 cities and municipalities by trained survey teams, each consisting of a physician, a key interviewer, an examiner, and a medical laboratory technician. The data quality and the nationwide representativeness of the KiGGS study were subjected to intensive internal and external quality controls (see Kamtsiuris et al. 2007 for details). For the 0–10 age group, parents reported about their children (proxy interviews), while for the 11–17 age group, both parents and adolescents were interviewed. The KiGGS baseline survey was conducted between May 2003 and May 2006. The baseline comprised a total sample of 17,641 children and adolescents; 68% of all baseline participants took part in the follow-up survey, KiGGS Wave 1. Wave 1 was carried out between June 2009 and June 2012 as standardized telephone interviews.

For the following analyses, we looked at interpersonal-environmental factors within a total longitudinal sample of all *n*_w_ = 11,691 children and adolescents aged between 0 and 17 years old (*n*_w_ = weighted; unweighted *n*_uw_ = 11,677). Of the 11,992 participants that took part in both the baseline study and Wave 1315 had to be excluded due to missing data for soft drink consumption. In addition to this, we also included individual lifestyle in addition to interpersonal-environmental correlates for a weighted sample of *n*_w_ = 5197 participants aged between 11 and 17 years old (*n*_uw_ = 4114), as individual lifestyle factors such tobacco or alcohol consumption were only available from 11 years on.

### Measures

Main outcome: In KiGGS wave 1, sweetened beverage consumption was measured with two items assessing the current frequency of children’s and adolescents’ consumption and the number of glasses consumed per day or per week (Mensink et al. 2018). The question on frequency referred to “the last few weeks” and read “How often do you/does your child drink sweetened beverages like cola, lemonade, ice tea, or malt beer? Sweetened beverages refer to sugar-sweetened and artificially sweetened beverages”. In this article, we have explicitly used the phrase “sweetened beverages” when referring to our own data (in contrast to “SSBs” that were explicitly examined in several studies referred to in Introduction and Discussion). These questions and our analyses explicitly excluded the consumption of water, milk, fruit, and vegetable juices. The available ordinal categories were “daily”, “not daily but at least once a week”, “less often than once a week”, and “never” (Mensink et al. 2018). Depending on the answer to the frequency question, participants were either asked for the average number of glasses of sweetened beverages that they drank per day or per week. It was explained to participants that “a glass” represents 200 ml (6.76 oz); answers such as “half a glass per day” or “half a glass per week” (i.e. 100 ml/3.38 oz) were also possible. A dichotomous variable was created from these self-reports, indicating if participants drank sweetened beverages at least once a week (dummy value 1) or less often than once a week (dummy value 0). In addition, a continuous variable of consumed glasses per day for every child or adolescent was computed. The continuous variable was set to zero if participants answered “never” or “less often than once a week” to the question on consumption frequency.

All potential correlates were recorded as part of the KiGGS baseline survey; these included age and gender as individual variables. Health-related lifestyle behaviours were also assessed. Namely, self-reported smoking (current consumption: yes vs. no), alcohol consumption (has participant ever consumed alcohol: yes vs. no), media consumption (spending time on a computer, a mobile phone, a games console or a television; categorized into terciles of usage time), consumption of fruit, cooked vegetables, salad, and other raw vegetables (assessed using a food frequency questionnaire, which also referred to ‘‘the last few weeks''; the total of 10 frequency categories were grouped together into three categories [< 3 times a week; 3–4 times a week; > 4 times a week]) and physical activity (How often is participant physically active in his or her free time, getting sweaty or out of breath; grouped into three categories [never; 1–2 times a month up to 3–5 times a week; and daily]).

In addition, interpersonal information (parents’ socio-economic status, immigration background, number of siblings, age of mother, and parental tobacco consumption) and environmental information (size of town of residence, region of residence) were assessed. The socioeconomic status (SES) of the participant’s parents was defined as “low”, “medium”, or “high” based on an established and validated index (Winkler and Stolzenberg 1999). This multidimensional aggregated index comprises the dimensions of parents’ education (school education and professional qualifications), income (net household income of all household members), and occupational status. In accordance with national standards, immigration background was deduced from the participant’s country of birth and nationality and country of birth of the participant’s parents (Schenk et al. 2008). It was also assessed whether children or adolescents lived together with siblings in the same household. Participant’s parents were asked if they were currently smokers. The size of the participant’s town of residence was determined using official data on political-administrative municipality sizes. This distinguishes between rural areas, small towns, medium-sized towns, and metropolitan areas (Robert-Koch-Institut 2008). Participants region of residence was assigned to the western (former West Germany—Federal Republic of Germany) or the eastern federal states (former East Germany—German Democratic Republic, including Berlin).

### Statistical analyses

We examined longitudinal associations using the individual and interpersonal-environmental variables from the baseline study (KiGGS Baseline) and the measures of sweetened beverage consumption from the follow-up study (KiGGS Wave 1). Variables were analysed using descriptive, bivariate, and multivariate methods. For the descriptive and bivariate analyses, data were weighted to adjust for dropout and make the longitudinal sample representative for the population in terms of gender, age, region, and migration background (weight made available by the Robert Koch Institute). Because the mother’s age is more strongly associated with the consumption of sweetened beverages than the father’s age, only mother’s age was included as a predictor in the analyses to avoid collinearity.

Outcome variables of interest were both dichotomous (sweetened beverage consumption ≥ once a week or not) and continuous (number of consumed glasses of sweetened beverages per day). These different variable types were analysed in separate models, to ensure the robustness of the analyses. Pearson Chi-Squared tests were used for the bivariate analyses with the dichotomous outcome variable; one-way analysis of variance were used with the continuous outcome variable.

For the multivariate analyses, multivariate logistic regressions with individual and interpersonal-environmental variables were conducted to analyse associations with the dichotomous variable. After that we employed general linear modelling with the continuous measure of sweetened beverage consumption. Given the categorical nature of our independent variables, we specified contrasts comparing the respective category to a reference category for each variable. We specified four predefined model variants for both outcome variables: in Model 1, we analysed sociocultural variables for the full sample. Models 2–4 comprised the subgroup of all adolescents aged eleven or above, because most behaviour-related variables were not assessed in younger age groups. The predefined level of significance was set at *p* < 0.05. All analyses were conducted using SPSS version 25.

## Results

The KiGGS Wave 1 data show that of all 6–24-year-olds in Germany, 59% consumed sweetened beverages regularly, that is, at least once a week, 23% reported daily consumption. On average, all children and adolescents of the total sample drank 1.12 ± 2.08 glasses per day, which is equal to approximately 224 ml ± 416 ml per day (median = 0.29 (58 ml), min–max: 0–20 (4 l) glasses per day).

### Individual correlates of sweetened beverage consumption

Soft drink consumption increased with age; particularly between the ages of 3–7 and 7–11, reaching a plateau later on. Older and male adolescents consumed a particularly high volume of sweetened beverages with particularly high frequency. In terms of individual lifestyle, above average consumption of sweetened beverages in KiGGS Wave 1 was accompanied by other behaviours that are detrimental to health such as high consumption of tobacco, alcohol, and media, as well as insufficient consumption of fruit and vegetables in the baseline study. Additionally, adolescents who reported a high level of physical activity drank significantly higher volumes of sweetened beverages more frequently (Table 1).

**Table 1:** Individual correlates of sweetened beverage consumption in German children and adolescents (nationwide representative KiGGS study, Germany, 2003–2012)

Correlates	*n*	Proportion (column %)	Sweetened beverage consumption ≥ once a week (%)^a^	*p* value	Sweetened beverage consumption (200 ml glasses/day)^b^	*p* value
*Individual variables*						
Age at baseline (and mean age at follow-up [min–max])				*p* < 0.001		*p* < 0.001
0 to < 3 years old (AM: 7.1; [6–9])	1604	13.7	36.2		0.41	
3 to < 7 years old (AM: 10.6; [8–13])	2447	20.9	52.7		0.65	
7 to < 11 years old (AM: 14.5; [12–17])	2443	20.9	66.0		1.27	
11 to < 14 years old (AM: 18.1; [16–20]	2037	17.4	67.5		1.47	
14–17 years old (AM: 21.6; [19–24])	3160	27.0	64.5		1.50	
Gender				*p* < 0.001		*p* < 0.001
Female	5728	49.0	50.9		0.80	
Male	5963	51.0	66.7		1.43	
Tobacco consumption				*p* < 0.001		*p* < 0.001
Yes	996	19.3	71.5		2.08	
No	4160	80.7	64.1		1.34	
Alcohol consumption				*p* = 0.124		*p* < 0.001
Yes	3282	63.9	64.8		1.58	
No	1854	36.1	67.0		1.31	
Media consumption				*p* < 0.001		*p* < 0.001
Low	1645	32.8	57.4		1.13	
Medium	1628	32.5	68.4		1.28	
High	1743	34.7	71.9		2.01	
Fruit consumption				*p* < 0.001		*p* < 0.001
< 3 times a week	1537	30.7	71.4		1.77	
3–4 times a week	903	18.0	68.3		1.57	
> 4 times a week	2564	51.2	60.3		1.22	
Consumption of cooked vegetables				*p* = 0.002		*p* < 0.001
< 3 times a week	3523	70.6	66.7		1.54	
3–4 times a week	916	18.4	61.8		1.34	
> 4 times a week	552	11.1	60.9		1.00	
Consumption of raw vegetables				*p* < 0.001		*p* < 0.001
< 3 times a week	2348	47.0	67.5		1.72	
3–4 times a week	1127	22.5	65.3		1.32	
> 4 times a week	1523	30.5	61.2		1.14	
Physical activity				*p* < 0.001		*p* < 0.001
Never	489	9.5	58.6		1.49	
1–2 times a month up to 3–5 times a week	3493	68.2	65.2		1.40	
Daily	1139	22.2	69.6		1.75	

Notes: See Electronic Supplementary Material (ESM) for complete table. Displayed are the bivariate associations of the respective variables with the dichotomous outcome variable and the continuous outcome variable

The data was weighted with the official longitudinal weight to adjust for dropout and make the longitudinal sample representative for the German population in terms of gender, age, region, and migration background. Cases are rounded

*AM* arithmetic mean

^a^*p* values refer to the Pearson Chi-squared test

^b^*p* values refer to the analyses one-way analysis of variance (ANOVA) which for variables with two categories yields the same result as an independent samples *t* test with equal variances assumed. *p* values refer to the analyses of variance (ANOVA)

### Interpersonal and environmental correlates of sweetened beverage consumption

The descriptive analyses identify clearly defined demographic subgroups with a particularly high level of consumption, for example, adolescents with a low social status drank high volumes of sweetened beverages particularly often. Other interpersonal and environmental factors that also showed bivariate associations with an increased consumption of sweetened beverages were the presence of siblings in the household, the mother’s age being comparatively high, tobacco consumption of one or both parents, and living in a more rural area (Table 2).

**Table 2:** Interpersonal and environmental correlates of sweetened beverage consumption in German children and adolescents (nationwide representative KiGGS study, Germany, 2003–2012)

Correlates	*n*	Proportion (column %)	Sweetened beverage consumption ≥ once a week (%)^a^	*p* value	Sweetened beverage consumption (glasses/day)^b^	*p* value
*Interpersonal and environmental variables*						
Socioeconomic status				*p* < 0.001		*p* < 0.001
Low	3903	33.9	65.9		1.42	
Medium	5139	44.7	59.5		1.14	
High	2464	21.4	46.4		0.62	
Immigration background				*p* < 0.001		*p* = 0.031
None	8802	75.5	58.1		1.15	
One parent	937	8.0	57.4		0.99	
Both parents	1922	16.5	63.8		1.06	
Siblings at home				*p* < 0.001		*p* = 0.023
None	2466	22.1	54.0		1.04	
One or more	8682	77.9	60.5		1.15	
Mother’s age				*p* < 0.001		*p* < 0.001
< 30 years old	1236	10.8	51.1		0.69	
≥ 30 years old	10,257	89.2	59.6		1.17	
Tobacco consumption of one or both parents				*p* < 0.001		*p* < 0.001
Yes	5938	51.2	62.5		1.30	
No	5660	48.4	55.0		0.94	
Size of administrative municipality				*p* = 0.429		*p* = 0.003
Rural	2129	18.2	60.0		1.26	
Small town	3220	27.5	59.1		1.13	
Medium-sized town	3405	29.1	57.9		1.10	
Metropolitan area	2937	25.1	59.2		1.04	
Region of residence				*p* = 0.005		*p* = 0.016
West (former FRG)	9702	83.0	59.5		1.10	
East (former GDR)	1989	17.0	56.1		1.23	

Notes: See Electronic Supplementary Material (ESM) for complete table. Displayed are the bivariate associations of the respective variables with the dichotomous outcome variable and the continuous outcome variable. The data was weighted with the official longitudinal weight to adjust for dropout and make the longitudinal sample representative for the German population in terms of gender, age, region, and migration background. Cases are rounded

^a^*p* values refer to the Pearson Chi-squared test

^b^*p* values refer to the one-way analysis analyses of variance (ANOVA) which for variables with two categories yields the same result as an independent samples *t* test with equal variances assumed. *p* values refer to the analyses of variance (ANOVA)

### Multivariate analyses of sweetened beverage consumption

The bivariate correlations described above were analysed from multiple perspectives using regression models. These analyses showed that most of the independent variables previously identified as significant correlates of consumption, remained significant after adjusting for potential confounders. For example, if the consumption of sweetened beverages is operationalized as a dichotomous variable then age, gender, tobacco and media consumption, and social status remain significant correlates in an adjusted logistic regression model (Table 3).

**Table 3:** Logistic Regression Models for sweetened beverage consumption in German children and adolescents (nationwide representative KiGGS study, Germany, 2003–2012)

Correlates	Children and adolescents			Adolescents								
	Whole cohort			Only children aged 11 and over								
	Model 1			Model 2			Model 3			Model 4		
	Adjusted OR [95% CI]		*p* value	Adjusted OR [95% CI]		*p* value	Adjusted OR [95% CI]		*p* value	Adjusted OR [95% CI]		*p* value
*Individual variables*												
Age												
0 to < 3 years old	Reference											
3 to < 7 years old	2.00	[1.76; 2.29]	*p* < 0.001									
7 to < 11 years old	3.56	[3.10; 4.09]	*p* < 0.001									
11 to < 14 years old	3.76	[3.24; 4.37]	*p* < 0.001	Reference						Reference		
14–17 years old	3.15	[2.73; 3.65]	*p* < 0.001	0.82	[0.71; 0.95]	*p* = 0.007				0.78	[0.66; 0.94]	*p* = 0.007
Gender												
Female	Reference			Reference						Reference		
Male	2.06	[1.90; 2.22]	*p* < 0.001	3.02	[2.61; 3.49]	*p* < 0.001				2.77	[2.38; 3.23]	*p* < 0.001
Tobacco consumption^a^												
Yes							1.34	[1.09; 1.64]	*p* = 0.006	1.48	[1.19; 1.83]	*p* < 0.001
No							Reference			Reference		
Alcohol consumption^b^												
Yes							0.84	[0.73; 0.98]	*p* = 0.023	0.98	[0.81; 1.17]	*p* = 0.783
No							Reference		Reference			
Media consumption												
Low							Reference			Reference		
Medium							1.54	[1.31; 1.81]	*p* < 0.001	1.29	[1.09; 1.53]	*p* = 0.003
High							1.77	[1.49; 2.11]	*p* < 0.001	1.28	[1.06; 1.54]	*p* = 0.010
Fruit consumption												
< 3 times a week							Reference		Reference			
3–4 times a week							1.03	[0.82; 1.28]	*p* = 0.829	1.15	[0.91; 1.45]	*p* = 0.235
> 4 times a week							0.76	[0.64; 0.91]	*p* = 0.002	0.89	[0.74; 1.07]	*p* = 0.206
Consumption of raw vegetables^c^												
< 3 times a week							Reference			Reference		
3–4 times a week							0.92	[0.76; 1.10]	*p* = 0.345	0.95	[0.79; 1.15]	*p* = 0.593
> 4 times a week							0.80	[0.67; 0.94]	*p* = 0.007	0.87	[0.73; 1.04]	*p* = 0.123
Physical activity												
Never							Reference			Reference		
1–2 times a month up to 3–5 times a week							1.43	[1.12; 1.83]	*p* = 0.004	1.12	[0.86; 1.44]	*p* = 0.403
Daily							1.89	[1.43; 2.50]	*p* < 0.001	1.27	[0.95; 1.71]	*p* = 0.108
*Interpersonal and environmental variables*												
Socioeconomic status												
Low	Reference			Reference						Reference		
Medium	0.82	[0.74; 0.92]	*p* < 0.001	0.83	[0.69; 1.01]	*p* = 0.068				0.86	[0.71; 1.05]	*p* = 0.148
High	0.53	[0.47; 0.60]	*p* < 0.001	0.50	[0.41; 0.63]	*p* < 0.001				0.55	[0.44; 0.68]	*p* < 0.001
Tobacco consumption of one or both parents												
Yes	1.25	[1.15; 1.35]	*p* < 0.001	1.17	[1.01; 1.35]	*p* = 0.038				1.09	[0.94; 1.26]	*p* = 0.275
No	Reference			Reference								
Region of residence												
West (former FRG)	Reference			Reference						Reference		
East (former GDR, including Berlin)	0.98	[0.90; 1.07]	*p* = 0.706	0.80	[0.68; 0.93]	*p* = 0.004				0.78	[0.66; 0.92]	*p* = 0.002
*N*	10,981			3589			3589			3589		
Cox and Snell *R*^2^	0.092			0.083			0.035			0.095		
Nagelkerkes *R*^2^	0.123			0.113			0.048			0.129		

Notes: Only significant correlates are displayed. See Electronic Supplementary Material (ESM) for complete table. Outcome: Sweetened beverage consumption (as dichotomous variable: at least once a week = 1 versus never or less often than once a week = 0)

Note that self-reported lifestyle behaviours were only available for children 11 years and older

^a^Current consumption

^b^Has child or adolescent ever consumed alcohol?

^c^Including salad

A similar picture emerges when general linear modelling is used: in addition to the correlates previously mentioned as significant, low to moderate consumption of vegetables, high degree of physical activity, and parental tobacco consumption are also significantly associated with sweetened beverage consumption 6 years later (see supplementary materials for Table 4 and for additional analyses stratified by age below and above 11 years).

## Discussion

**Fig. 1:**
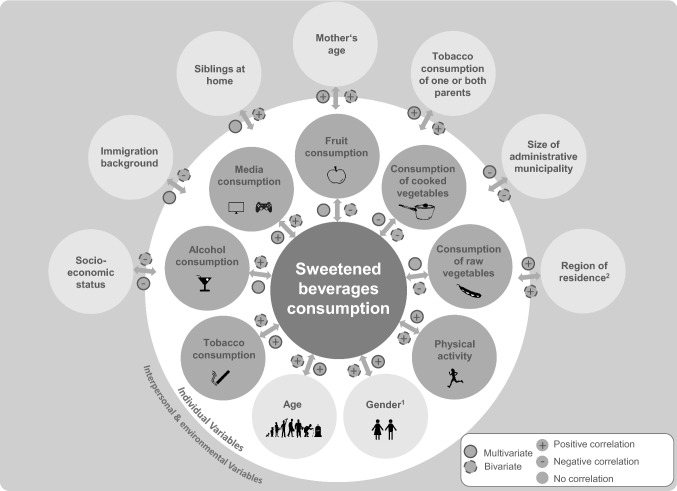
Individual and interpersonal-environmental correlates of sweetened beverage consumption (bivariate and multivariate associations with the independent variable “glasses per day”) among children and adolescents in Germany. *Notes*: Associations between behaviour-related variables and sweetened beverage consumption refer to the cohort of children aged 11 and over (dark grey circular area); all other associations refer to the whole cohort (light grey circular area). See method section for details. If one of the categories showed a significant contrast to the respective reference category, significance is displayed. ^1^Male. ^2^Former German Democratic Republic (Nationwide representative KiGGS study, Germany, 2003–2012)

Drinking sweetened beverages is a normal part of everyday life for the majority of German children, adolescents, and post-adolescents—59% of all 6–24-year-olds in Germany consume sweetened beverages regularly, 23% even daily. This high level of consumption is often accompanied by other unhealthy lifestyle behaviours including a high level of tobacco and media consumption as well as low fruit and vegetable intake. Older and male adolescents with a low social status drink a higher than average amount of sweetened beverages. Just being male is associated with drinking about 1 more 200 ml glass of sweetened beverage—per day. Figure 1 provides a synoptic depiction of the findings on correlates of sweetened beverage consumption.

### Principal findings and contribution to the current state of research

While a number of our results are in line with previous findings from other Western countries, they also deliver important new insights and observations. As previously reported for other countries, in Germany we have also found that sweetened beverage consumption increases with age (Denney-Wilson et al. 2009; Hennessy et al. 2015; Vereecken et al. 2005). However, our data show a plateau in late adolescence, while other sources tend to report a continuous increase until post-adolescence (Bere et al. 2008; Pettigrew et al. 2015; Scully et al. 2017; Van Ansem et al. 2014). A deciding factor in the higher level of consumption observed in adolescents compared with children is their increasing autonomy and greater disposable income (Bere et al. 2008; Pettigrew et al. 2015; Scully et al. 2017; Van Ansem et al. 2014). Comparable to findings from other countries, boys in our sample consume sweetened beverage more frequently and in larger amounts than girls (Bere et al. 2008; Bjelland et al. 2011; Denney-Wilson et al. 2009; Park et al. 2012, 2013; Scully et al. 2017; Sdrali et al. 2010; Vereecken et al. 2005). Although boys generally require a higher energy intake than girls, this increased energy demand is nevertheless best fulfilled with healthy food. Other reasons discussed for higher levels of SSB consumption in boys include a lower degree of health consciousness, less concern with own appearance, and gender specific socialisation (Vereecken et al. 2005).

This study enters completely new territory by conducting a comprehensive analysis addressing the “big four” (Khaw 2008) classic characteristics of health-relevant lifestyle: smoking, alcohol, nutrition, and physical activity. Previous studies have only addressed individual health-relevant lifestyle factors related to the consumption of SSBs. A few studies have reported a positive link between SSB consumption and tobacco and alcohol consumption (Park et al. 2012, 2013). Other studies have found a correlation between SSB and media consumption (Mazarello Paes et al. 2015; Park et al. 2012; Scully et al. 2017). Individual studies have also observed this behaviour among primary school children (Lopez et al. 2012; Olafsdottir et al. 2014) and even in pre-school aged children (Garnett et al. 2013; Olafsdottir et al. 2014). One particularly interesting study differentiated television consumption by weekday and found a particularly strong correlation with SSB consumption on weekends (Pettigrew et al. 2015). This could be due to influence of food advertising on adolescent viewers as well as longer TV exposition times on weekends, which lead to interruption of physiological food regulation, e.g. satiety cues (Scully et al. 2017; Verzeletti et al. 2009). For example, snacking while using a computer is also accompanied by a significant increase in SSB consumption (Verzeletti et al. 2009). Findings on the relationship between SSB consumption and eating fruits and vegetables are inconsistent (Park et al. 2012; Ranjit et al. 2010; Scully et al. 2017). In contrast to our German study population, data from the USA and Greece point to a negative correlation between SSB consumption and physical activity (Park et al. 2012; Sdrali et al. 2010).

Eating habits such as SSB consumption do not evolve in isolation, but within a micro-environment, which is primarily influenced by interpersonal relationships with family members, and within a macro-environment (Schneider et al. 2017; Van Ansem et al. 2014). We have already explored in detail how, under certain circumstances, these micro- and macro-environments can result in what is known as an obesogenic environment (Schneider et al. 2017). The home environment represents an important part of a person’s micro-environment. Parents are their children’s nutritional gatekeepers, their knowledge and choices determine what their children eat (e.g. Dallacker et al. 2016; 2018). Children have little control over purchases and generally eat the food that is available at home (Lopez et al. 2012; Elfhag et al. 2008).

One particularly striking finding from our data is that the consumption of sweetened beverages is about twice as high for children from low-status families compared to children from higher status families. This classic social gradient—usually measured using the socio-economic status of the parents—has been well documented (Bere et al. 2008; Garnett et al. 2013; Hebden et al. 2013; Lopez et al. 2012; Lundeen et al. 2018; Mazarello Paes et al. 2015; Park et al. 2014; Scully et al. 2017; Sdrali et al. 2010; Totland et al. 2013; Van Ansem et al. 2014). Parents could use SSBs as coping mechanisms for emotional discomfort in their children. Garnett et al. (2013) suggest another parental motive for this finding, namely that the introduction of SSBs may be a mechanism for coping with food insecurity and that SSBs are used as a means to quell behavioural agitation. Although the argument of limited economic resources (Vereecken et al. 2005) may play a subjective role for parents when shopping, de facto, tap water, bottled carbonated water, non-carbonated mineral water, or other alternatives such as tea and flavoured calorie-free water are generally cheaper than SSBs.

Interestingly, it seems that mothers play a more important role in SSB consumption than fathers—a stronger correlation was found between SSB consumption and the mother’s behaviour than with that of the father (Bjelland et al. 2011); this finding holds equally true for both, girls and boys (Elfhag et al. 2008). Furthermore, estimations regarding the availability of SSBs within the family home tend to correspond more closely between mother and child than between father and child, indicating that mothers have better knowledge than fathers of the food selection actually available in the home (Totland et al. 2013). Other publications also emphasize the role of the mother as a role model (Bjelland et al. 2011; Denney-Wilson et al. 2009) for their children’s dietary behaviour. A study on regulatory food parenting has shown that mothers play a greater role in monitoring, regulating, and controlling their children’s eating habits than fathers (Melbye et al. 2016). We did not replicate previous findings in which a higher level of SSB consumption was observed among the children of younger mothers (Garnett et al. 2013; Mazarello Paes et al. 2015); on the contrary, in our data set, having an older mother was associated with higher consumption. As in previous studies, our data are also inconclusive with regard to the significance of siblings (Mazarello Paes et al. 2015; Park et al. 2014) and the region of residence (Garnett et al. 2013).

### Limitations and strengths

When interpreting the KiGGS study, it is important to consider the definition of sweetened beverages, a potential social desirability bias, and residual confounding factors.

There is currently no internationally agreed standard definition for SSBs (Mazarello Paes et al. 2015). As in most studies, the method we use to operationalize our data also meant that other drinks such as sweetened milk, sweetened coffee-based drinks, and sweetened vegetable juice were excluded. Likewise, as done in many comparable studies, we also excluded various types of sweetened fruit juice and sports drinks. Flavoured sports beverages usually contain large amounts of added sugar (Ranjit et al. 2010); however, studies have shown that adolescent consumers and their parents tend to subjectively view the health effects of this special type of sweetened beverage differently than other soft drinks, resulting in positive associations between consuming sports beverages and several healthy lifestyle practices (Hennessy et al. 2015; Ranjit et al. 2010).

Unfortunately, the food frequency questionnaire used here did not allow for the extraction of specific data regarding the consumption of calorie-reduced drinks (e.g. low calorie, practically calorie-free, “diet” soft drinks). However, given that in Germany more than 80% of all soft drinks consumed by adolescents are classic SSBs (Mensink et al. 2018), this limitation likely has little impact on our findings. Furthermore, several of the health risks mentioned in the Introduction, such as development of metabolic syndrome, type 2 diabetes, or the displacement of milk and calcium in the diet are still equally applicable when sugar-free versions of soft drinks are consumed. Lastly, prospective cohort studies have demonstrated a positive correlation between the consumption of artificial sweeteners and weight gain. It is proposed that the regular exposure to sweet flavours, regardless of their origin, leads to a persisting preference for sweet things and thus a preference for high-calorie food. This is accompanied by the fact that artificial sweeteners (e.g. aspartame) also function as appetite stimulants (Elfhag et al. 2007). It should be mentioned that the role of artificially sweetened beverages for the development of obesity is controversially discussed, particularly in industry-funded studies (e.g. Peters et al. 2014).

With a total of 16 independent variables, we were able to consider more variables than most other comparable studies. Nevertheless, a risk remains that the regressions presented here may be affected by residual confounding factors from unmeasured but relevant influences. We also tested for multicollinearity: the highest correlation between the independent variables (included in Table 3 and in Table 4 (see supplementary materials), models 2–4) was *r* = .56 and thus well below common thresholds for multicollinearity.

Finally, as with any epidemiological questionnaire-based study, typical social desirability bias cannot be completely eliminated in the reporting of eating habits. The main strengths of this study are its large, representative sample size, the extensive quality assurance measures implemented in the field work, its longitudinal design, and the comprehensive consideration of health-relevant lifestyle practices.

### Conclusions

Health and health inequalities start to develop very early in life due to the complex interactions of cumulative risks and resources at the individual, meso-, and macro-level (Seabrook and Avison 2012). Sweetened beverages are one piece of this puzzle. We show that various dimensions of an unhealthy lifestyle occur accumulatively. Our findings call for complex, multi-level interventions. Next to the role of individual risk behaviours such as smoking or low vegetable intake related to sweetened beverage consumption, our data also highlight the central role played by a person’s social context—their home environment in particular. Because dietary habits are formed in childhood, food skills and the learning of healthy choices are particularly important early in life. For example, one recent study reports that the participation of young people in the preparation of family meals was the top predictor of food skills later in life (Seabrook et al. 2019). Interventions need to aim not only at children and adolescents, but especially at their parents and the (interpersonal) environments inhabited by children and adolescents (Park et al. 2014). In addition to reducing the consumption of unhealthy SSBs, interventions also need to address other unhealthy lifestyle habits that go hand in hand with drinking SSBs, such as eating little fruits and vegetables, media use, and physical activity (Bogart et al. 2017).

## Electronic supplementary material

Below is the link to the electronic supplementary material.

Supplementary material 1 (DOCX 34 kb)
