# Supplementary material for: Relations between sweetened beverage consumption and individual, interpersonal, and environmental factors: a 6-year longitudinal study in German children and adolescents
Source: Int J Public Health. 2020 Jun 20;65(5):559–70. doi: 10.1007/s00038-020-01397-0 (PMC7360664; doi:10.1007/s00038-020-01397-0)
Supplement: Supplementary file 1 — Supplementary material 1 (DOCX 34 kb) [file 38_2020_1397_MOESM1_ESM.docx]

**Journal Name: International Journal of Public Health**

**Article title: Relations between sweetened beverage consumption and individual, interpersonal, and environmental factors: a 6-year longitudinal study in German children and adolescents**

Table S1: Bivariate correlations between interpersonal and environmental correlates of sweetened beverage consumption investigated in this study (n = 3,589)

| Variables | 1. | 2. | 3. | 4. | 5. | 6. | 7. | 8. | 9. | 10. | 11. | 12. | 13. | 14. | 15. | 16. |
| --- | --- | --- | --- | --- | --- | --- | --- | --- | --- | --- | --- | --- | --- | --- | --- | --- |
|  |  |  |  |  |  |  |  |  |  |  |  |  |  |  |  |  |
| 1. Age | – |  |  |  |  |  |  |  |  |  |  |  |  |  |  |  |
| 1. Gender | -0.039* | – |  |  |  |  |  |  |  |  |  |  |  |  |  |  |
| 1. Tobacco consumption^1^ | 0.324** | -0.048** | – |  |  |  |  |  |  |  |  |  |  |  |  |  |
| 1. Alcohol consumption^2^ | 0.560** | -0.022 | 0.313** | – |  |  |  |  |  |  |  |  |  |  |  |  |
| 1. Media consumption | -0.041* | 0.240** | -0.015 | 0.046** | – |  |  |  |  |  |  |  |  |  |  |  |
| 1. Fruit consumption | -0.063** | -0.111** | -0.118** | -0.059** | -0.160** | – |  |  |  |  |  |  |  |  |  |  |
| 1. Consumption of cooked vegetables | 0.024 | -0.009 | -0.030 | -0.005 | -0.069** | 0.141** | – |  |  |  |  |  |  |  |  |  |
| 1. Consumption of raw vegetables^3^ | -0.018 | -0.114** | -0.065** | -0.044** | -0.154** | 0.325** | 0.190** | – |  |  |  |  |  |  |  |  |
| 1. Physical activity | -0.179** | 0.238** | -0.099** | -0.112** | -0.011 | 0.118** | 0.022 | 0.076** | – |  |  |  |  |  |  |  |
| 1. Socio-economic status | 0.024 | 0.033* | -0.056** | -0.008 | -0.124** | 0.097** | 0.132** | 0.111** | 0.026 | – |  |  |  |  |  |  |
| 1. Immigration background | -0.018 | 0.014 | -0.034* | -0.102** | 0.087** | 0.012 | -0.035* | 0.024 | -0.032 | -0.157** | – |  |  |  |  |  |
| 1. Siblings at home | -0.044** | 0.023 | -0.084** | -0.068** | -0.085** | 0.029 | 0.029 | 0.033* | 0.048** | 0.018 | 0.040* | – |  |  |  |  |
| 1. Mother’s age | 0.243** | 0.043** | 0.067** | 0.156** | -0.028 | -0.061** | 0.085** | 0.039* | -0.032 | 0.223** | -0.083** | -0.037* | – |  |  |  |
| 1. Tobacco consumption of one or both parents | -0.007 | -0.026 | 0.131** | 0.313** | 0.119** | -0.088** | -0.059** | -0.089** | -0.020 | -0.202** | 0.029 | -0.083** | -0.118** | – |  |  |
| 1. Size of administrative municipality | 0.008 | 0.006 | -0.005 | -0.069** | 0.067** | -0.023 | 0.076** | 0.007 | -0.045** | 0.113** | 0.203** | -0.081** | 0.047** | 0.054** | – |  |
| 1. Region of residence | 0.024 | -0.027 | 0.088** | 0.084** | 0.033 | 0.107** | -0.065** | -0.073** | -0.042* | 0.021 | -0.184** | -0.135** | -0.217** | 0.052** | -0.092** | – |

Notes: ** p < .01; * p < .05 (2-tailed)

1) Current consumption.

2) Has child or adolescent ever consumed alcohol?

3) Including salad.

Table S2: Interpersonal and environmental correlates of sweetened beverage consumption in German children aged below 11 years (weighted n = 6,495). Displayed are the bivariate associations of the respective variables with the dichotomous outcome variable and the continuous outcome variable.

|  | Correlates | |  | | n | | proportion (column %) | sweetened beverage consumption ≥ once a week^a^ | p-value | sweetened beverage consumption (glasses/day)^b^ | p-value |  |  |
| --- | --- | --- | --- | --- | --- | --- | --- | --- | --- | --- | --- | --- | --- |
| Interpersonal & environmental variables | | | | | | | | | | | | | |
|  | |  | |  | |  |  |  |  |  |  | |  |
|  | | Socioeconomic status | |  | |  |  |  | p < 0.001 |  | p < 0.001 | |  |
|  | | Low | |  | | 2,088 | 32.6 % | 62.3 % |  | 1.15 |  | |  |
|  | | Medium | |  | | 2,811 | 43.9 % | 53.9 % |  | 0.81 |  | |  |
|  | | High | |  | | 1,505 | 23.5 % | 41.0 % |  | 0.44 |  | |  |
|  | | Missing | |  | | 91 |  |  |  |  |  | |  |
|  | |  | |  | |  |  |  |  |  |  | |  |
|  | | Immigration background | |  | |  |  |  | p < 0.001 |  | p = 0.541 | |  |
|  | | None | |  | | 4,779 | 73.9 % | 51.8 % |  | 0.83 |  | |  |
|  | | One parent | |  | | 607 | 9.4 % | 52.3 % |  | 0.76 |  | |  |
|  | | Both parents | |  | | 1,078 | 16.7 % | 62.3 % |  | 0.86 |  | |  |
|  | | Missing | |  | | 30 |  |  |  |  |  | |  |
|  | |  | |  | |  |  |  |  |  |  | |  |
|  | | Siblings at home | | | | |  |  | p < 0.001 |  | p < 0.001 | |  |
|  | | None | |  | | 1,469 | 23.8 % | 45.5 % |  | 0.60 |  | |  |
|  | | One or more | |  | | 4,715 | 76.2 % | 56.4 % |  | 0.89 |  | |  |
|  | | Missing | |  | | 311 |  |  |  |  |  | |  |
|  | |  | |  | |  |  |  |  |  |  | |  |
|  | | Mother’s age | |  | |  |  |  | p = 0.011 |  | p < 0.001 | |  |
|  | | <30 years old | |  | | 1,211 | 18.8 % | 50.3 % |  | 0.67 |  | |  |
|  | | ≥30 years old | |  | | 5,231 | 81.2 % | 54.3 % |  | 0.86 |  | |  |
|  | | Missing | |  | | 52 |  |  |  |  |  | |  |
|  | |  | |  | |  |  |  |  |  |  | |  |
|  | | Tobacco consumption of one or both parents | | | | | |  | p < 0.001 |  | p < 0.001 | |  |
|  | | Yes | |  | | 3,203 | 49.6 % | 58.3 % |  | 0.98 |  | |  |
|  | | No | |  | | 3,256 | 50.4 % | 48.8 % |  | 0.67 |  | |  |
|  | | Missing | |  | | 36 |  |  |  |  |  | |  |
|  | |  | |  | |  |  |  |  |  |  | |  |
|  | | Size of administrative municipality | | | | |  |  | p = 0.060 |  | p = 0.332 | |  |
|  | | Rural (< 5,000 inhabitants) | |  | | 1,131 | 17.4 % | 56.0 % |  | 0.91 |  | |  |
|  | | Small town (5,000 to <20,000 inhabitants) | |  | | 1,747 | 26.9 % | 53.8 % |  | 0.81 |  | |  |
|  | | Medium-sized town (20,000 to <100,000 inhabitants) | |  | | 1,920 | 29.6 % | 51.2 % |  | 0.81 |  | |  |
|  | | Metropolitan area (≥100,000 inhabitants) | |  | | 1,697 | 26.1 % | 54.5 % |  | 0.81 |  | |  |
|  | |  | |  | |  |  |  |  |  |  | |  |
|  | | Region of residence | | | | | |  | p = 0.926 |  | p = 0.095 | |  |
|  | | West (former FRG) | |  | | 5,454 | 84.0 % | 53.6 % |  | 0.81 |  | |  |
|  | | East (former GDR, including Berlin) | |  | | 1,041 | 16.0 % | 53.8 % |  | 0.91 |  | |  |
|  | |  | |  | |  |  |  |  |  |  | |  |

Notes:

The data was weighted with the official longitudinal weight to adjust for dropout and make the longitudinal sample representative for the German population in terms of gender, age, region, and migration background. Cases are rounded.

^a^ p-values refer to the Pearson Chi-Squared Test.

^b^ p-values refer to the one-way analysis of variance (ANOVA) which for variables with two categories yields the same result as an independent samples t-test with equal variances assumed.

Table S3: Interpersonal and environmental correlates of sweetened beverage consumption in German children and adolescents aged 11 years and over (weighted n = 5,197). Displayed are the bivariate associations of the respective variables with the dichotomous outcome variable and the continuous outcome variable.

|  | Correlates | |  | | n | | proportion (column %) | sweetened beverage consumption ≥ once a week^a^ | p-value | sweetened beverage consumption (glasses/day)^b^ | p-value |  |  |
| --- | --- | --- | --- | --- | --- | --- | --- | --- | --- | --- | --- | --- | --- |
| Interpersonal & environmental variables | | | | | | | | | | | | | |
|  | |  | |  | |  |  |  |  |  |  | |  |
|  | | Socioeconomic status | |  | |  |  |  | p < 0.001 |  | p < 0.001 | |  |
|  | | Low | |  | | 1,816 | 35.6 % | 70.1 % |  | 1.72 |  | |  |
|  | | Medium | |  | | 2,328 | 45.6 % | 66.3 % |  | 1.55 |  | |  |
|  | | High | |  | | 959 | 18.8 % | 54.8 % |  | 0.89 |  | |  |
|  | | Missing | |  | | 94 |  |  |  |  |  | |  |
|  | |  | |  | |  |  |  |  |  |  | |  |
|  | | Immigration background | |  | |  |  |  | p = 0.889 |  | p = 0.046 | |  |
|  | | None | |  | | 4,023 | 77.4 % | 65.5 % |  | 1.53 |  | |  |
|  | | One parent | |  | | 329 | 6.3 % | 66.9 % |  | 1.40 |  | |  |
|  | | Both parents | |  | | 844 | 16.2 % | 65.6 % |  | 1.31 |  | |  |
|  | |  | |  | |  |  |  |  |  |  | |  |
|  | | Siblings at home | | | | |  |  | p = 0.613 |  | p = 0.005 | |  |
|  | | None | |  | | 997 | 20.1 % | 66.4 % |  | 1.68 |  | |  |
|  | | One or more | |  | | 3,967 | 79.9 % | 65.5 % |  | 1.44 |  | |  |
|  | | Missing | |  | | 232 |  |  |  |  |  | |  |
|  | |  | |  | |  |  |  |  |  |  | |  |
|  | | Mother’s age | |  | |  |  |  | p = 0.012 |  | p = 0.680 | |  |
|  | | <30 years old | |  | | 26 | 0.5 % | 88.5 % |  | 1.71 |  | |  |
|  | | ≥30 years old | |  | | 5025 | 99.5 % | 65.1 % |  | 1.49 |  | |  |
|  | | Missing | |  | | 146 |  |  |  |  |  | |  |
|  | |  | |  | |  |  |  |  |  |  | |  |
|  | | Tobacco consumption of one or both parents | | | | | |  | p = 0.003 |  | p < 0.001 | |  |
|  | | Yes | |  | | 2,735 | 53.2 % | 67.5 % |  | 1.66 |  | |  |
|  | | No | |  | | 2,404 | 46.8 % | 63.5 % |  | 1.29 |  | |  |
|  | | Missing | |  | | 57 |  |  |  |  |  | |  |
|  | |  | |  | |  |  |  |  |  |  | |  |
|  | | Size of administrative municipality | | | | |  |  | p = 0.818 |  | p = 0.038 | |  |
|  | | Rural (< 5,000 inhabitants) | |  | | 998 | 19.2 % | 64.6 % |  | 1.65 |  | |  |
|  | | Small town (5,000 to <20,000 inhabitants) | |  | | 1,473 | 28.3 % | 65.4 % |  | 1.51 |  | |  |
|  | | Medium-sized town (20,000 to <100,000 inhabitants) | |  | | 1,485 | 28.6 % | 66.5 % |  | 1.48 |  | |  |
|  | | Metropolitan area (≥100,000 inhabitants) | |  | | 1,240 | 23.9 % | 65.7 % |  | 1.35 |  | |  |
|  | |  | |  | |  |  |  |  |  |  | |  |
|  | | Region of residence | | | | | |  | p < 0.001 |  | p = 0.250 | |  |
|  | | West (former FRG) | |  | | 4,248 | 81.7 % | 67.2 % |  | 1.47 |  | |  |
|  | | East (former GDR, including Berlin) | |  | | 949 | 18.3 % | 58.7 % |  | 1.57 |  | |  |
|  | |  | |  | |  |  |  |  |  |  | |  |

Notes:

The data was weighted with the official longitudinal weight to adjust for dropout and make the longitudinal sample representative for the German population in terms of gender, age, region, and migration background. Cases are rounded.

^a^ p-values refer to the Pearson Chi-Squared Test.

^b^ p-values refer to the one-way analysis of variance (ANOVA) which for variables with two categories yields the same result as an independent samples t-test with equal variances assumed.
